# Relationship between serum lipid levels and the immune microenvironment in breast cancer patients: a retrospective study

**DOI:** 10.1186/s12885-022-09234-8

**Published:** 2022-02-14

**Authors:** Wataru Goto, Shinichiro Kashiwagi, Yuri Kamei, Chika Watanabe, Naoki Aomatsu, Katsumi Ikeda, Yoshinari Ogawa, Kosei Hirakawa, Masaichi Ohira

**Affiliations:** 1 Department of Breast Surgical Oncology, Osaka City General Hospital, 2-13-22 Miyakojima-hondori, Miyakojima-ku, Osaka, 534-0021 Japan; 2 Department of Breast and Endocrine Surgery, Osaka City University Graduate School of Medicine, 1-4-3 Asahi-machi, Abeno-ku, Osaka, 545-8585 Japan; 3 Department of Gastrointestinal Surgery, Osaka City University Graduate School of Medicine, 1-4-3 Asahi-machi, Abeno-ku, Osaka, 545-8585 Japan

**Keywords:** Breast cancer, Tumor immune microenvironment, Lipid metabolism, Recurrence

## Abstract

**Background:**

Therapeutic agents for dyslipidaemia, in particular statins, have been recently reported to suppress growth and metastasis of breast cancer. However, the predictive value of lipid control in breast cancer patients has not been discussed sufficiently. In addition, though immunometabolism is a relatively novel approach for tumour immunotherapy, the relationship between lipid metabolism and immune status has not been well documented. We therefore investigated the effects of lipid metabolism on antitumour immune response and cancer prognosis.

**Methods:**

Except for patients with ductal carcinoma in situ, 938 patients treated with curative surgery were examined. The correlation between treatment for dyslipidaemia or serum lipid levels and clinicopathological features, including the prognosis, was evaluated retrospectively. Also, we stratified these results by intrinsic subtype of breast cancer, menopause, and type of therapeutic agents for dyslipidaemia. Moreover, neutrophil- to-lymphocyte ratio (NLR) and tumour-infiltrating lymphocytes (TILs) were used as indicators of systemic and local immune status, respectively.

**Results:**

Of 194 patients treated for dyslipidaemia, recurrence-free survival (RFS) and overall survival (OS) did not differ significantly between users of drugs for dyslipidaemia and non-users (*p* = 0.775 and *p* = 0.304, log-rank, respectively). Among postmenopausal, hormone receptor (HR)-positive/human epidermal growth factor receptor 2 (HER2)-negative breast cancer patients treated for dyslipidaemia, the good serum lipid control group had significantly better RFS (*p* = 0.014, log-rank), lower postoperative NLR (*p* = 0.012), and higher TILs in resected tissues (*p* = 0.024) than the poor control group. Multivariate analysis showed that postoperative serum lipid levels were a risk factor for recurrence (hazard ratio = 4.722, 95% confidence interval 1.006–22.161, *p* = 0.049).

**Conclusions:**

Good control of serum lipid metabolism may improve the tumour immune microenvironment and prognosis in postmenopausal HR-positive/HER2-negative breast cancer patients.

**Supplementary Information:**

The online version contains supplementary material available at 10.1186/s12885-022-09234-8.

## Background

Regulation and improvement of the tumour immune microenvironment are widely recognized to play an important role in cancer therapy [1, 2]. Immunometabolism is a relatively new field in tumour immunotherapy; the regulation of metabolism can affect the tumour immune microenvironment and enhance antitumour immunity [3–6]. For example, although some meta-analyses found a significant risk of breast cancer in women with type 2 diabetes mellitus [7, 8], the use of metformin in the treatment of type 2 diabetes mellitus has been reported to reduce cancer-related mortality [9]. Metformin’s antitumour activity has been associated with its modulation of components of the tumour immune microenvironment such as tumour-associated macrophages, myeloid-derived suppressor cells, and T-cells [3, 10, 11].

On the other hand, in the case of lipid metabolism, a positive association has been reported between obesity and the risk of breast cancer among postmenopausal, hormone receptor (HR)-positive women [12, 13]. Furthermore, some previous studies also have shown that the obesity prior to and after diagnosis were predictive of breast cancer recurrence and death, especially in HR positive type [14, 15]. Additionally, although epidemiologic evidence shows no association between the usage of statins, therapeutic agents for dyslipidaemia, and reduced incidence of breast cancer [16–18], it supports a protective effect of statins on reducing breast cancer recurrence or mortality [19]. Also, some studies suggested that lipophilic statins, but not hydrophilic statins, improved the prognosis in breast cancer patients [20, 21].

Several basic researches have shown the effects of therapeutic agents for dyslipidaemia, mainly statins, on breast cancer cells. Statins suppress cancer cell proliferation by inducing cell cycle arrest through the regulation of cyclin-dependent kinase 4/6 (CDK4/6) or cyclin D1 [22, 23]. Statins have also been shown to exert anti-angiogenic effects through a reduction in hypoxia-induced vascular endothelial growth factor secretion and expression of vascular endothelial growth factor receptor 2 and matrix metalloproteinase-9 in endothelial cells [24, 25]. Furthermore, statins have been shown to reduce the invasiveness and metastatic potential of breast cancer cells [26, 27]. However, limited information is clinically available on the systemic and local immune response to statin treatment in breast cancer patients. In this study, we evaluated the correlation between improvement in lipid metabolism along with antitumour immune response and prognosis in breast cancer patients treated with curative surgery.

## Materials and methods

### Patients

We analysed data from patients treated at the Osaka City General Hospital (Osaka, Japan) from the period between April 2010 and March 2017. A total of 1018 patients were diagnosed with breast cancer and underwent curative surgery during this period. We excluded 80 patients with ductal carcinoma in situ and included 938 breast cancer patients in this retrospective study. Postoperative adjuvant therapy was administered according to the intrinsic breast cancer subtype, and standard postoperative radiotherapy was administered to the remnant breast, if necessary. T and N factors as well as tumour stage were stratified based on the TNM Classification, UICC Seventh Edition [28]. Tumours were classified into intrinsic subtypes according to the immunohistochemical expression of the oestrogen receptor, progesterone receptor, and human epidermal growth factor receptor 2 (HER2). The HR-positive group comprised oestrogen receptor-positive and/or progesterone receptor-positive patients. All patients were examined for continuation of agents for dyslipidaemia before undergoing mastectomy or breast-conserving surgery.

This present study is exploratory research. First, we divided patients into two groups according to the use of agents for dyslipidaemia, and examined whether treatments for dyslipidaemia were associated with prognosis in breast cancer patients, as has been reported. Next, we evaluated serum lipid levels of patients treated with the drugs for dyslipidaemia before and after surgery. We defined patients whose postoperative serum lipid levels were in the normal range as the good control group and patients with high lipid levels as the poor control group, and examined whether lipid control by dyslipidaemia medication was associated with prognosis. We also performed subgroup analysis by menopause and intrinsic breast cancer subtypes to identify the group in which lipid control was most associated with recurrence. Finally, we examined the relationship between lipid control and clinicopathological features, including immune status, in the identified group.

We set two survival end points: (I) overall survival (OS) was defined as the time from surgery until death from any cause, and (II) recurrence-free survival (RFS) was defined as freedom from all loco-regional and distant recurrences. All patients were followed up by physical examination, blood tests, ultrasonography, computed tomography, and bone scintigraphy. The median follow-up period for the assessment of OS was 4.0 years (range, 0.1–8.9 years) and for RFS was 3.6 years (range, 0.1–8.9 years).

### Blood sample analysis

Preoperative blood samples were obtained within one week before surgery, and postoperative blood samples were obtained annually. We evaluated serum lipid levels, including total cholesterol (categorized as low (< 150 mg/dL), normal (150–219 mg/dL), or high (> 219 mg/dL) and triglyceride (categorized as low (< 50 mg/dL), normal (50–149 mg/dL), or high (> 149 mg/dL)). The differential white blood cell count was analysed using a Coulter LH 750 Hematology Analyzer (Beckman Coulter, Brea, CA, USA). In this study, the neutrophil-to-lymphocyte ratio (NLR) was considered to be the systemic immune response indicator. NLR was calculated from the blood sample by dividing the absolute neutrophil count by the absolute lymphocyte count.

### Histopathological evaluation

In this study, the density of tumour-infiltrating lymphocytes (TILs) was considered to be the local immune response indicator. TILs can be used as indicators of the tumour microenvironment and are important in predicting clinical outcomes in breast cancer [29, 30]. In this study, tumour specimens were used to evaluate TILs density. The assessment of haematoxylin and eosin (HE)-stained TILs was based on the criteria described by the International TILs Working Group 2014 [31]. TILs were defined as the infiltrating lymphocytes within the stromal compartment close to the tumour. To evaluate TILs, four fields of view in HE-stained areas were selected, and the percentage of TILs in each field was measured microscopically at 400× magnification. HE-stained TILs were defined to be high if TILs occupied > 10% and low when TILs occupied ≤ 10% of the HE-stained field of view (Supplementary Fig. S1).

### Statistical analysis

Statistical analysis was performed using the JMP13 software program (SAS Institute, Cary, NC, USA). Associations among variables were analysed using chi-square (χ^2^) or Fisher’s exact test, as appropriate. OS and RFS were estimated using the Kaplan–Meier method and the log-rank test. Univariate and multivariate hazard ratios (HRs) and 95% confidence intervals (CIs) were computed using the Cox proportional hazards model. Receiver operating characteristic curve analysis was performed to select the most appropriate cut-off value for NLR. A *p* value < 0.05 was considered significant.

## Results

### The relationship between treatments for dyslipidaemia and prognosis in all breast cancer patients

Differences in clinicopathological features due to treatments for dyslipidaemia are presented in Table 1. Of 938 breast cancer patients, 194 (20.7%) received treatment for dyslipidaemia (Supplementary Fig. S2). Age (years) ranged from 20 to 94 (mean, 60.1; median, 61; standard deviation (SD), 13.4; inter quartile range (IQR), 61). Significant association was observed between patients who were administered the drug for dyslipidaemia and older age (*p* < 0.001), menopause (*p* < 0.001), and diabetes mellitus (*p* < 0.001). Only 89 (9.5 %) patients received neoadjuvant chemotherapy (NAC) in this study, and more patients treated for dyslipidemia did not received NAC significantly than those without treatment for dyslipidemia (*p* = 0.004).

**Table 1:** Differences in clinicopathological features due to treatments for dyslipidemia

	Treatment for dyslipidemia (*n* = 938)		
	No (*n* = 744)	Yes (*n* = 194)	*p* value
Age at diagnosis (year)			
≤61 / >61	438 (58.9%) / 306(41.1%)	47 (24.2%) / 147(75.8%)	*p*<0.001
Menopause			
Pre / Post	267 (35.9%) / 477(64.1%)	14 (7.2%) / 180(92.8%)	*p*<0.001
Diabetes			
Negative / Positive	693 (93.2%) / 51(6.8%)	142 (73.2%) / 52(26.8%)	*p*<0.001
Tumor size (cm)			
≤2 / >2	422 (56.7%) / 322(43.3%)	108 (55.7%) / 86(44.3%)	*p* = 0.808
Lymph node status			
Negative / Positive	492 (66.1%) / 252(33.9%)	138 (71.1%) / 56(28.9%)	*p* = 0.199
Neoadjuvant chemotherapy			
No / Yes	663 (89.1%) / 81(10.9%)	186 (95.9%) / 8(4.1%)	*p* = 0.004
Intrinsic subtype			
Luminal / Luminal-HER /	513 (68.9%) / 96(12.9%) /	131 (67.5%) / 23(11.9%) /	*p* = 0.861
HER2-enruch / TNBC	51 (6.9) / 84(11.3%)	16 (8.2) / 24(12.4%)	

*HER2* human epidermal growth factor receptor 2, *TNBC* triple-negative breast cancer

**Fig. 1:**
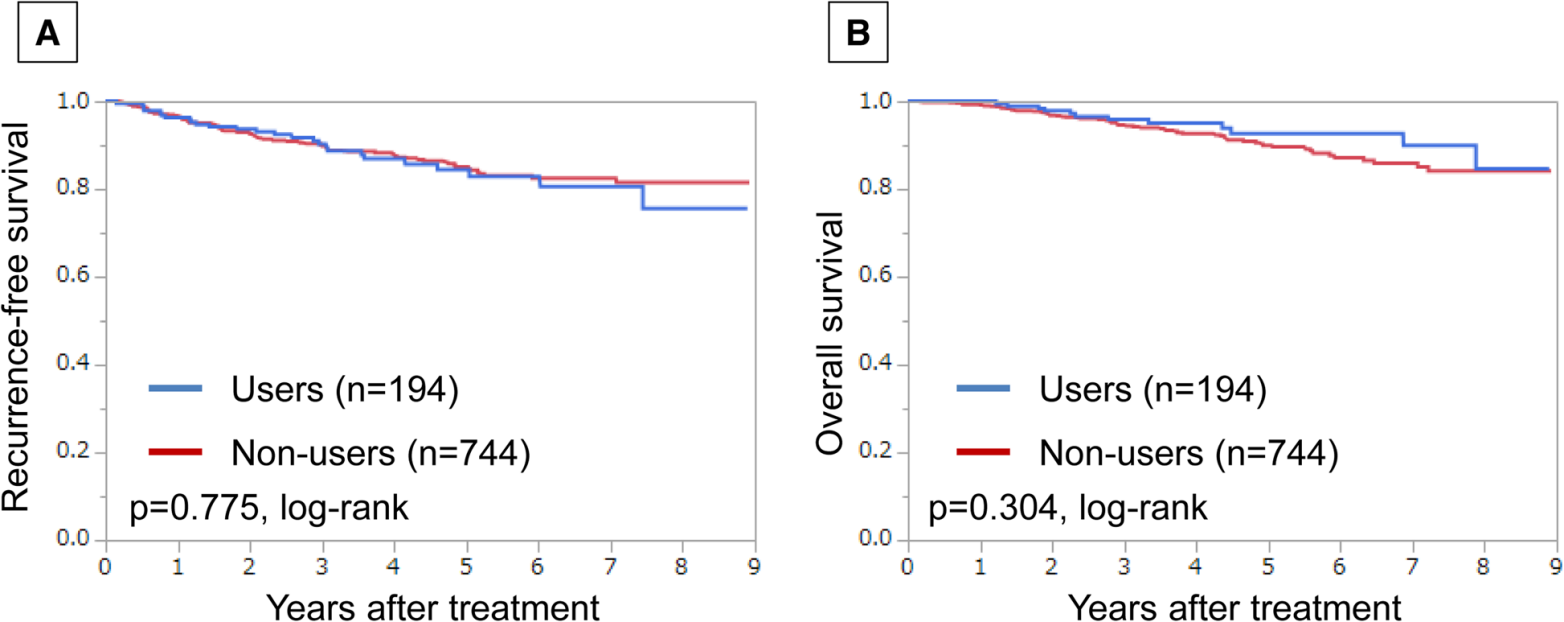
Recurrence-free survival (RFS) and overall survival (OS) in all patients based on users or non-users of drugs for dyslipidaemia. Estimated Kaplan-Meier curves of RFS (**a**) and OS (**b**)

In all patients, no significant differences in RFS or OS were observed due to the use of agents for dyslipidaemia (*p* = 0.775, *p* = 0.304, log-rank, respectively) (Fig. 1). Additionally, we investigated the prognostic value of administration of the drug(s) for dyslipidaemia in each breast cancer subtype; no associations between the treatment for dyslipidaemia and RFS or OS were found in each intrinsic subtype (Supplementary Figs. S3 and S4).

### The relationship between lipid control and prognosis in patients treated for dyslipidaemia

**Fig. 2:**
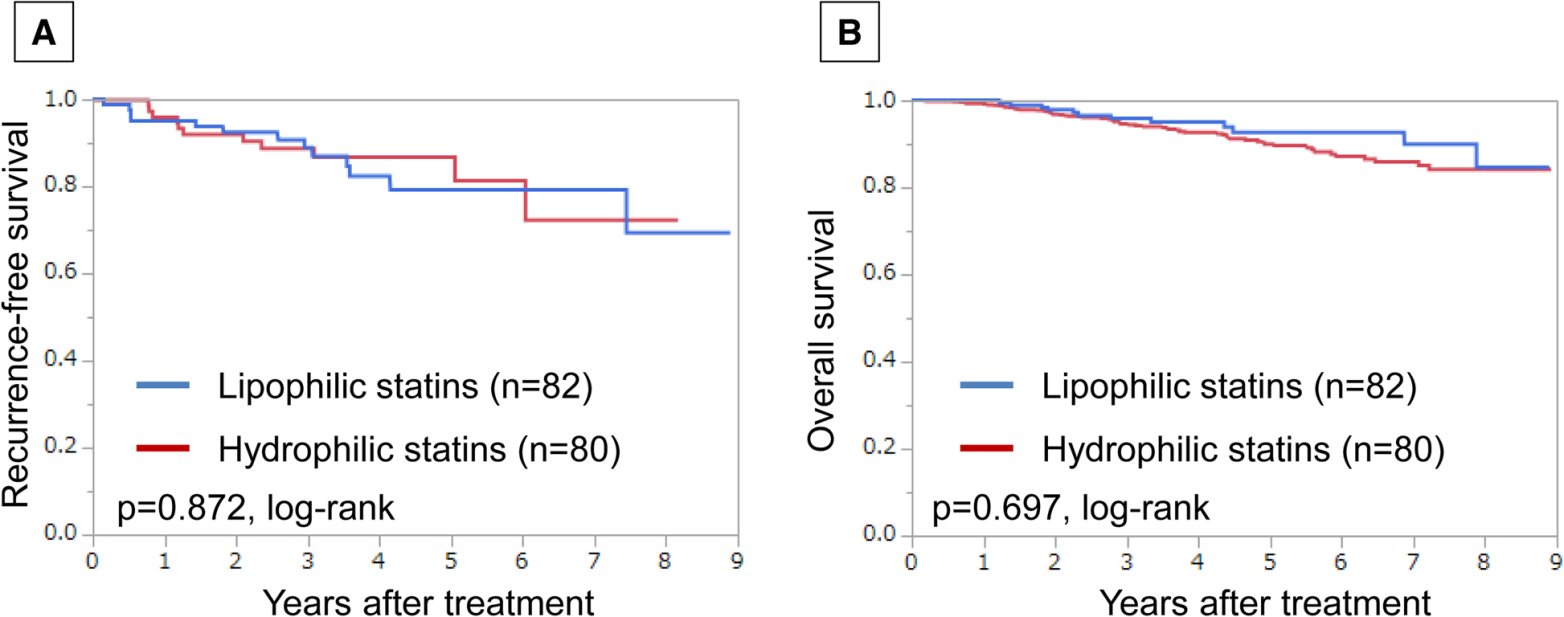
Recurrence-free survival (RFS) and overall survival (OS) in patients treated for dyslipidemia based on statin type. Estimated Kaplan-Meier curves of RFS (**a**) and OS (**b**)

In 194 patients treated for dyslipidaemia, the following anti-dyslipidaemia drugs were administered: pravastatin (29 cases), simvastatin (3 cases), fluvastatin (2 cases), atorvastatin (57 cases), pitavastatin (20 cases), rosuvastatin (51 cases), ezemitib (8 cases), bezafibrate (2 cases), fenofibrate (4 cases), ethyl icosapentate (2 cases), and elastaze (1 case). Eighty-two patients (42.3%) were treated with lipophilic statins and 80 patients (41.2%) with hydrophilic statins. The remaining 32 patients were either taking non-statin drugs or there are insufficient drug-related data for them. There was no significant difference in RFS or OS between lipophilic and hydrophilic statins (*p* = 0.872, *p* = 0.697, log-rank, respectively) (Fig. 2).

**Fig. 3:**
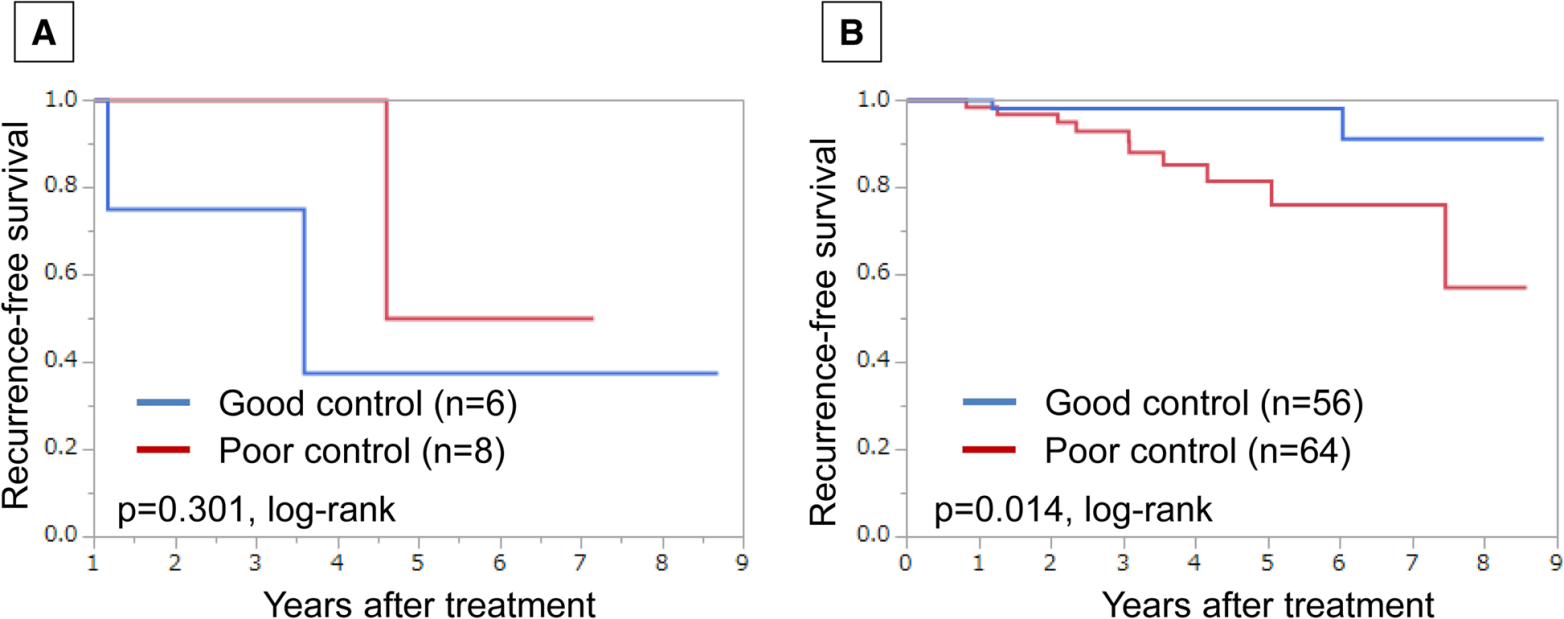
Recurrence-free survival (RFS) using Kaplan-Meier method in hormone receptor (HR)-positive/human epidermal growth factor receptor 2 (HER2)-negative patients treated for dyslipidaemia based on control of serum lipid levels. Premenopausal patients (**a**) and postmenopausal patients (**b**)

We investigated the prognostic value of serum lipid control by treatment for dyslipidaemia in each breast cancer intrinsic subtypes, and in only patients with HR-positive/HER2-negative breast cancer, the good control group had significantly better RFS than the poor control group (*p* = 0.045, log-rank) (Supplement Figs. S5 and S6). Because there was no death with luminal-HER2 breast cancer, statistical analysis of OS was not performed. We also classified patients with HR-positive/HER2-negative breast cancer according to menopausal status, and found that only postmenopausal patients showed significantly better RFS in the good control group compared with the poor control group (*p* = 0.014, log-rank) (Fig. 3).

### Analyses of postmenopausal patients with HR-positive/HER2-negative breast cancer treated for dyslipidaemia

We examined the correlation between clinicopathological features and serum lipid control of 120 postmenopausal patients with HR-positive/HER2-negative breast cancer treated for dyslipidaemia (Supplement Fig. S7) (Table 2). Age (years) ranged from 47 to 90 (mean, 65.6; median, 65.5; SD, 7.0; IQR, 69). Body mass index (kg/m^2^) ranged from 17.4 to 36.5 (mean, 25.7; median, 25.1; standard deviation, 5.2; IQR, 25.1). Good lipid control group had significantly good serum lipid levels before surgery (*p* = 0.006). NLR was determined for every sample, and the NLR cut-off value for serum lipid control was 1.65 (area under the curve: 0.63; sensitivity: 76.6%; specificity: 46.4%) (Supplementary Fig. S8). Good lipid control group had significantly good serum lipid levels before surgery (*p* = 0.006). Patients with good lipid control also showed significantly lower postoperative NLR than poor lipid control group (*p* = 0.012).

**Table 2:** Differences in clinicopathological features due to serum lipid control

	Serum lipid control (*n* = 120)		
	Poor (*n* = 64)	Good (*n* = 56)	*p* value
Age at diagnosis (year)			
≤65 / >65	21(32.8%) / 43(67.2%)	21(37.5%) / 35(62.5%)	*p* = 0.702
BMI (kg/m^2^)			
≤25.1 / >25.1	28(44.4%) / 35(55.6%)	32(57.1%) / 24(42.9%)	*p* = 0.200
Diabetes			
Negative / Positive	46(71.9%) / 18(28.1%)	39(69.6%) / 17(30.4%)	*p* = 0.842
Adjuvant therapy received			
No / Yes	17(26.6%) / 47(73.4%)	14(25.0%) / 42(75.0%)	*p* = 0.845
Serum lipid levels (preoperative)			
High / Low, normal	43(67.2%) / 21(32.8%)	23(41.1%) / 33(58.9%)	*p* = 0.006
Tumor size (cm)			
≤2 / >2	37(57.8%) / 27(42.2%)	35(62.5%) / 21(37.5%)	*p* = 0.709
Lymph node status			
Negative / Positive	42(65.6%) / 22(34.4%)	43(76.8%) / 13(23.2%)	*p* = 0.228
NLR (postoperative)			
High / Low	49(76.6%) / 15(23.4%)	30(53.6%) / 26(46.4%)	*p* = 0.012
TILs			
Low / High	45(73.8%) / 16(26.2%)	38(69.1%) / 17(30.9%)	*p* = 0.681

*BMI* body mass index, *NLR* neutrophil-to-lymphocyte ratio, *TILs* tumor-infiltrating lymphocytes

Moreover, we investigated the correlation between clinicopathological features and preoperative serum lipid levels (Supplementary Table S1). Patients with good preoperative lipid levels were significantly associated with high TILs density (*p* = 0.024). However, preoperative serum lipid levels were not associated with long RFS (*p* = 0.748, log-rank) (Supplementary Fig. S9). In addition, there was no significant relationship between TILs density and preoperative NLR (*p* = 0.612).

Postoperative NLR (HRs = 2.573×10^9^, 95%CI: 2.868–unparsable, *p* = 0.002) and serum lipid control (HRs = 5.534, 95%CI: 1.448–36.136, *p* = 0.010) were significantly correlated with RFS in univariate analyses. Multivariate analyses showed that good serum lipid control was an independent prognostic factor for recurrence (HRs = 4.722, 95%CI: 1.006–22.161, *p* = 0.049) (Table 3).

**Table 3:** Univariate and multivariate analyses with respect to RFS in postmenopausal HR-positive/HER2-negative breast cancer treated for dyslipidemia

	Univariate analysis			Multivariate analysis		
	Hazard ratio	95% CI	*p*-value	Hazard ratio	95% CI	*p*-value
Age at diagnosis (>65)	1.034	0.324-3.882	0.957			
BMI (>25.1)	1.383	0.423-4.548	0.584			
Diabetes (negative)	1.348	0.398-6.115	0.649			
Adjuvant chemotherapy (-)	1.196	0.264-4.044	0.793			
Serum lipid level (preoperative) (Low, normal)	1.204	0.376-3.852	0.748			
Tumor size (>2)	2.010	0.640-6.803	0.230			
Lymph node (+)	1.862	0.549-5.854	0.302			
NLR (preoperative) (High)	2.595	0.684-16.898	0.175			
NLR (postoperative) (High)	2.573×10^9^	2.868-unparsable	0.002	2.087×10^9^	2.367-unparsable	0.992
Serum lipid control (Poor)	5.534	1.448-36.136	0.010	4.722	1.006-22.161	0.049
TILs (High)	2.229	0.608-8.153	0.218			

Values in parentheses are 95 per cent confidence intervals

*CI* confidence interval, *BMI* body mass index, *NLR* neutrophil-to-lymphocyte ratio, *TILs* tumor-infiltrating lymphocyte

In addition, 49 patients (40.8%) were taking lipophilic statins, and 48 patients (40.0%) were taking hydrophilic statins. The remaining 23 patients were either taking non-statin drugs or drug-related data were insufficient for them. RFS tended to be better in the lipophilic statin users than in the hydrophilic statin users (*p* = 0.162, log-rank) (Supplementary Fig. S10).

**Fig. 4:**
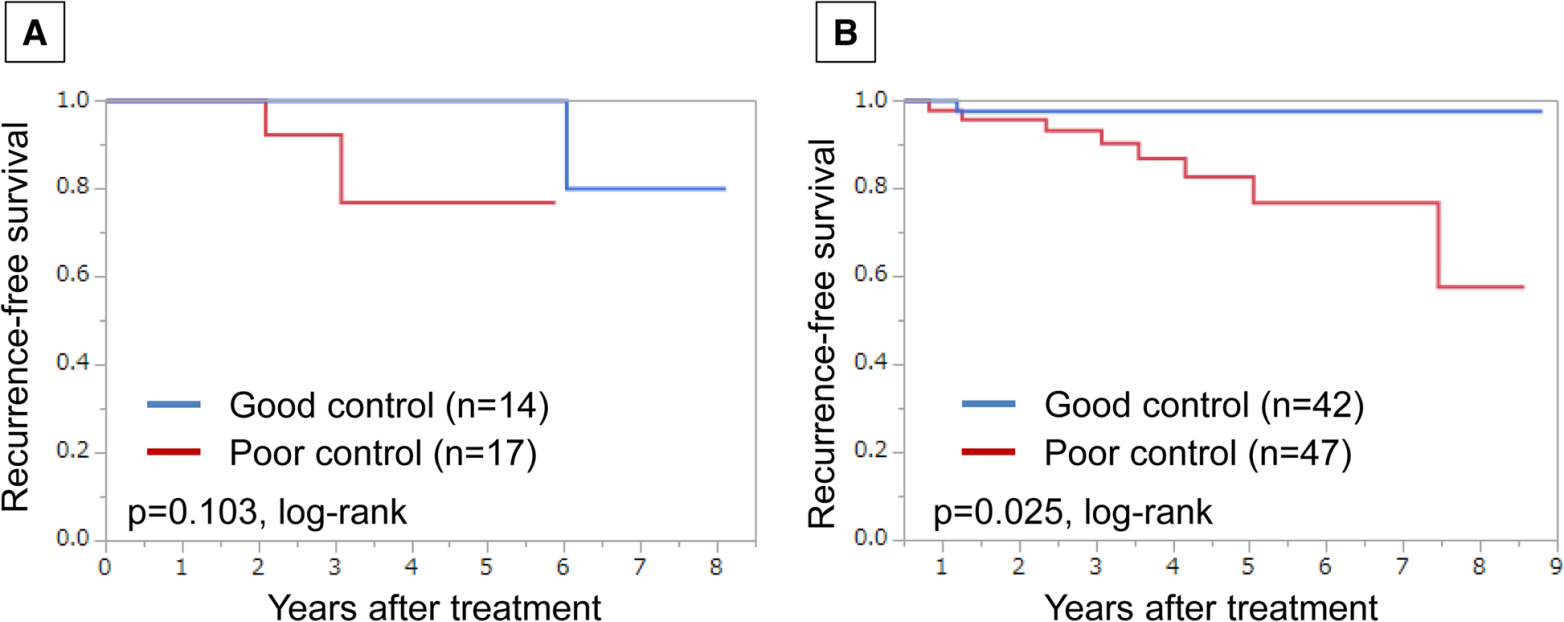
Recurrence-free survival (RFS) using Kaplan-Meier method in postmenopausal hormone receptor (HR)-postive/human epidermal growth factor receptor 2 (HER2)-negative patients treated for dyslipidemia based on control of serum lipid levels. Patients without adjuvant endocrine therapy (**a**) and patients with adjuvant endocrine therapy (**b**)

Furthermore, in this study, we also classified postmenopausal HR-positive/HER2-negative breast cancer patients treated for dyslipidaemia by administration of Ais. Only patients treated with AIs as adjuvant endocrine therapy showed significantly better RFS in the good lipid control group (*p* = 0.025, log-rank) (Fig. 4).

## Discussion

In this study, we verified the predictive value of anti-dyslipidaemia drug administration for the progression of breast cancer in patients treated with curative surgery. We did not observe any significant association between the use of drug(s) for dyslipidaemia and breast cancer prognosis after surgery in any breast cancer patient. Statin administration has been found to be associated with lowered risk of breast cancer-related recurrence or mortality [21, 32–34]. However, some studies have also reported that statin use was not significantly associated with a reduction in cancer-specific mortality [35, 36]. These inconsistent results could be attributed to age and timing of drug administration, drug types, and whether curative surgery or adjuvant therapy is being performed. Snyder et al. had indicated that statins were preferentially taken by patients who made better healthcare choices, engaged in healthier behaviours, and had better breast cancer prognoses [37]. Therefore, various subgroup analyses are very important. Some previous studies indicated that the benefits of statins for recurrence or mortality in breast cancer were most strongly seen with lipophilic statins [38, 39]. In our study, a weak non-significant improvement in RFS was observed for postmenopausal HR-positive/HER2-negative breast cancer patients treated with lipophilic statins compared with patients treated with hydrophilic statins.

We also stratified patients treated for dyslipidaemia by intrinsic subtype of breast cancer. Classification of breast cancer intrinsic subtypes is clinically useful in obtaining prognostic information [40]. In the present study, postmenopausal HR-positive/HER2-negative breast cancer patients with good serum lipid control due to the agents for dyslipidaemia showed significantly longer RFS, and these patients also had significantly lower postoperative NLR. NLR has been demonstrated to be a marker of systemic immunity and prognostic factor for breast cancer [41, 42]. Therefore, these results suggest that good control of lipid metabolism by the drugs for dyslipidaemia may improve systemic immune activity and induce suppression of recurrence in postmenopausal HR-positive/HER2-negative breast cancer patients.

Preoperative lipid metabolism was not significantly related to recurrence; thus, the data suggests that it is more important to control serum lipid levels after curative surgery. Preoperative serum lipid levels also did not have any significant relationship with preoperative NLR. However, patients whose preoperative lipid levels were low or normal were significantly associated with high TILs density in their tumour specimen. TILs can be used to monitor the tumour immune microenvironment and are important in predicting clinical outcomes in many types of cancer [29, 43, 44]. Thus, lipid metabolism may be related to the local tumour immune microenvironment.

**Fig. 5:**
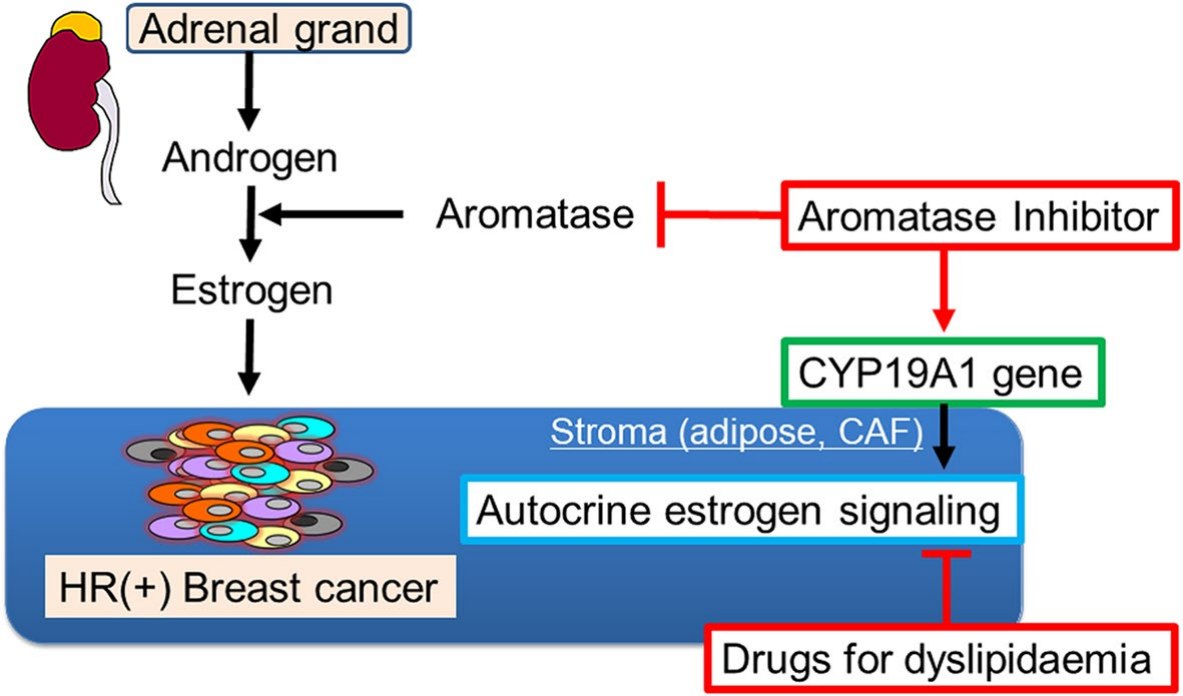
Schematic illustration of effects of drugs for dyslipidaemia. Aromatase inhibitor (AI) selects for acquired amplification of the *CYP19A1* (aromatase) gene and promotes local autocrine oestrogen signalling in patients with AI-resistant breast cancer. Aromatase is expressed from the stroma, including adipose tissue or cancer-associated fibroblasts; hence, serum lipid control by treatment for dyslipidaemia may modulate components of the tumour immune microenvironment and suppress the growth of AI-resistant breast cancer cells

In premenopausal HR-positive/HER2-negative breast cancer patients, there was no relationship between lipid control and recurrence. Unlike premenopausal breast cancer patients, androgens secreted from the adrenal grand in postmenopausal breast cancer patients are converted into oestrogen by aromatase mainly present in the stromal tissue. Hence, aromatase inhibitors (AIs) are recommended as adjuvant endocrine therapy for postmenopausal HR-positive breast cancer [45]. Cholesterol is the common precursor of sex hormones, including androstenedione and testosterone, the two substrates of the aromatase enzyme [46]; Thus, drugs for dyslipidaemia may act in the same way as AIs by lowering serum cholesterol levels. In this study, we found that postmenopausal HR-positive/HER2-negative breast cancer patients treated with AIs as adjuvant endocrine therapy showed significantly better RFS in the good lipid control group. Luca et al*.* revealed that AI treatment itself selected for acquired amplification of the *CYP19A1* (aromatase) gene and promoted local autocrine oestrogen signalling in patients with AI-resistant breast cancer [47]. Aromatase is expressed from the stroma, including adipose tissue; hence, serum lipid control by treatment for dyslipidaemia may modulate components of the tumour immune microenvironment, such as cancer-associated fibroblasts, and suppress the growth of AI-resistant breast cancer cells (Fig. 5) In the future, immunohistochemical analyses of primary or recurrent tumours and investigation of immune mechanisms may facilitate both the understanding of the relationship between lipid metabolism and the tumour immune microenvironment as well as the prediction of prognosis.

This study has some limitations: it is a single-centre retrospective and exploratory study. The sample size was small, and some confounders such as the dose- and time-dependence of the prognostic effect among patients treated for dyslipidaemia was unclear as it was not known whether patients were adherent to their medications. Further prospective multicentre studies are therefore needed to identify the strengths and weaknesses of our findings.

## Conclusions

This is the first study to demonstrate the clinical relationship between lipid metabolism and the tumour immune microenvironment in breast cancer patients. The findings of this study indicate that good control of serum lipid levels may improve the tumour immune microenvironment and predict a favourable outcome in postmenopausal HR-positive/HER2-negative breast cancer patients.

## Supplementary Information

**Additional file 1: Supplementary Figure S1.** Region of histopathological TILs evaluation. TILs were measured by examining the occupation ratio of immune cells present in the tumour stroma of hematoxylin and eosin stained specimens at 400x magnification. We determined that the proportion of TILs in the tumor stroma was> 10% as High (**a**) and ≤10% as Low (**b**)

**Additional file 2: Supplementary Figure S2.** Consort diagram. A total of 1018 patients were diagnosed with breast cancer and underwent curative surgery. We excluded 80 patients with ductal carcinoma in situ, and this retrospective study comprised 938 breast cancer patients. Of the 938 breast cancer patients, 194 were receiving treatment for dyslipidaemia.

**Additional file 3: Supplementary Figure S3.** Recurrence-free survival (RFS) using Kaplan-Meier method in patients based on users or non-users of drugs for dyslipidaemia with different intrinsic breast cancer subtype. Luminal (**a**), Luminal-human epidermal growth factor receptor 2 (HER2) (**b**), HER2-enrich (**c**) and triple-negative breast cancer (TNBC) (**d**).

**Additional file 4: Supplementary Figure S4.** Overall survival (OS) using Kaplan-Meier method in patients based on users or non-users of drugs for dyslipidemia with different intrinsic breast cancer subtype. Luminal (**a**), Luminal-human epidermal growth factor receptor 2 (HER2) (**b**), HER2-enrich (**c**) and triple-negative breast cancer (TNBC) (**d**).

**Additional file 5: Supplementary Figure S5.** Recurrence-free survival (RFS) using Kaplan-Meier method in patients treated for dyslipidemia based on control of serum lipid levels with different intrinsic breast cancer subtype. Luminal (**a**), Luminal-human epidermal growth factor receptor 2 (HER2) (**b**), HER2-enrich (**c**) and triple-negative breast cancer (TNBC) (**d**).

**Additional file 6: Supplementary Figure S6.** Overall survival (OS) using Kaplan-Meier method in patients treated for dyslipidemia based on control of serum lipid levels with different intrinsic breast cancer subtype. Luminal (**a**), HER2-enrich (**b**) and triple-negative breast cancer (TNBC) (**c**).

**Additional file 7: Supplementary Figure S7.** Consort diagram. Of the 120 postmenopausal patients with hormone receptor (HR)-positive/human epidermal growth factor receptor 2 (HER2)-negative breast cancer treated for dyslipidaemia, 56 were in good lipid control group.

**Additional file 8: Supplementary Figure S8.** Receiver operating characteristic curve analyses of the NLR in postmenopausal hormone receptor (HR)-postive/human epidermal growth factor receptor 2 (HER2)-negative breast cancer patients

**Additional file 9: Supplementary Figure S9.** Recurrence-free survival (RFS) and overall survival (OS) in postmenopausal hormone receptor (HR)-positive/human epidermal growth factor receptor 2 (HER2)-negative breast cancer patients treated for dyslipidaemia based on preoperative serum lipid levels. Estimated Kaplan-Meier curves of RFS (**a**) and OS (**b**).

**Additional file 10: Supplementary Figure S10.** Recurrence-free survival (RFS) using Kaplan-Meier method in postmenopausal hormone receptor (HR)-positive/human epidermal growth factor receptor 2 (HER2)-negative breast cancer patients treated for dyslipidaemia based on statin type.

**Additional file 11: Supplementary Table S1.** Differences in clinicopathological features due to preoperative serum lipid levels.

## Data Availability

The datasets used and/or analyzed during the current study are available from the corresponding author on reasonable request.

## Abbreviations

NLR: Neutrophil-to-lymphocyte ratio
TILs: Tumor-infiltrating lymphocytes
DFS: Recurrence-free survival
OS: Overall survival
HR: Hormone receptor
HER2: Human epidermal growth factor receptor-2
ER: Estrogen receptor
PgR: Progesterone receptor
HE: Hematoxylin and eosin
HRs: Hazard ratios
CI: Confidence interval
NAC: Neoadjuvant chemotherapy
BMI: Body mass index
AIs: Aromatase inhibitors
