# Supplementary material for: Relationship between serum lipid levels and the immune microenvironment in breast cancer patients: a retrospective study
Source: BMC Cancer. 2022 Feb 14;22:167. doi: 10.1186/s12885-022-09234-8 (PMC8842971; doi:10.1186/s12885-022-09234-8)
Supplement: Supplementary file 1 — Additional file 1: Supplementary Figure S1. Region of histopathological TILs evaluation. TILs were measured by examining the occupation ratio of immune cells present in the tumour stroma of hematoxylin and eosin stained specimens at 400x magnification. We determined that the proportion of TILs in the tumor stroma was> 10% as High (a) and ≤10% as Low (b) [file 12885_2022_9234_MOESM1_ESM.pdf]

Supplementary Fig. S1 Goto W. et al.

A

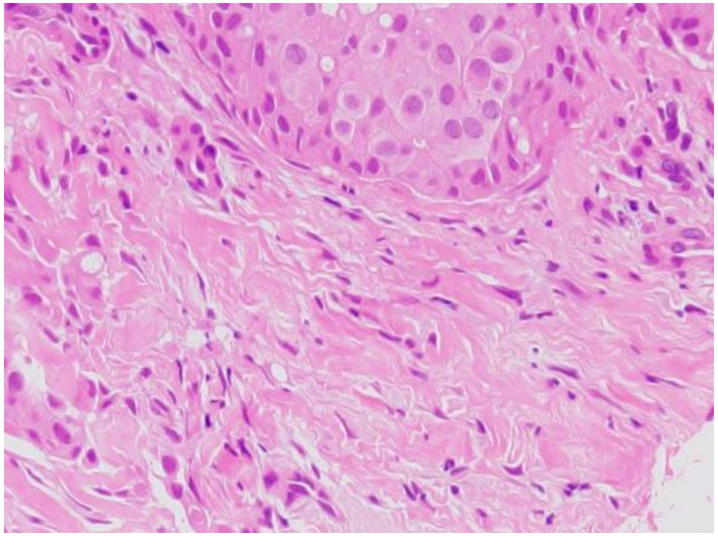

B

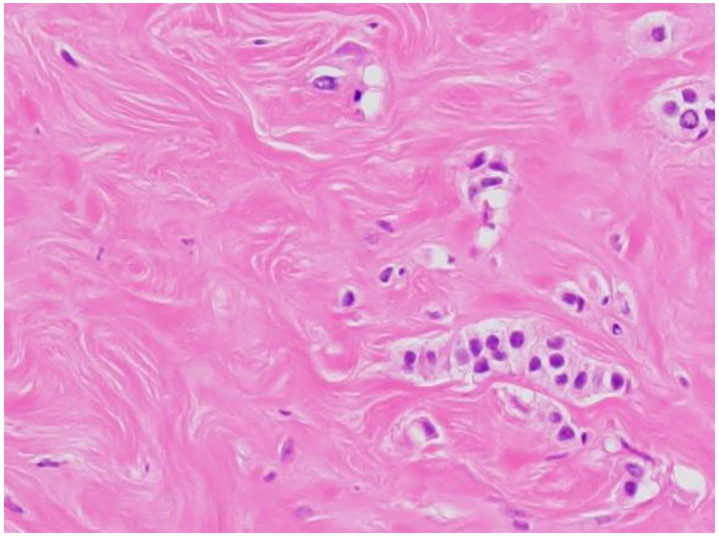

Supplementary Fig. S1 Region of histopathological TILs evaluation. TILs were measured by examining the occupation ratio of immune cells present in the tumour stroma of hematoxylin and eosin stained specimens at 400x magnification. We determined that the proportion of TILs in the tumor stroma was> 10% as High (**a**) and ≤10% as Low (**b**).
