# Supplementary material for: Relationship between serum lipid levels and the immune microenvironment in breast cancer patients: a retrospective study
Source: BMC Cancer. 2022 Feb 14;22:167. doi: 10.1186/s12885-022-09234-8 (PMC8842971; doi:10.1186/s12885-022-09234-8)
Supplement: Supplementary file 2 — Additional file 2: Supplementary Figure S2. Consort diagram. A total of 1018 patients were diagnosed with breast cancer and underwent curative surgery. We excluded 80 patients with ductal carcinoma in situ, and this retrospective study comprised 938 breast cancer patients. Of the 938 breast cancer patients, 194 were receiving treatment for dyslipidaemia. [file 12885_2022_9234_MOESM2_ESM.pdf]

Supplementary Fig. S2 Goto W. et al.

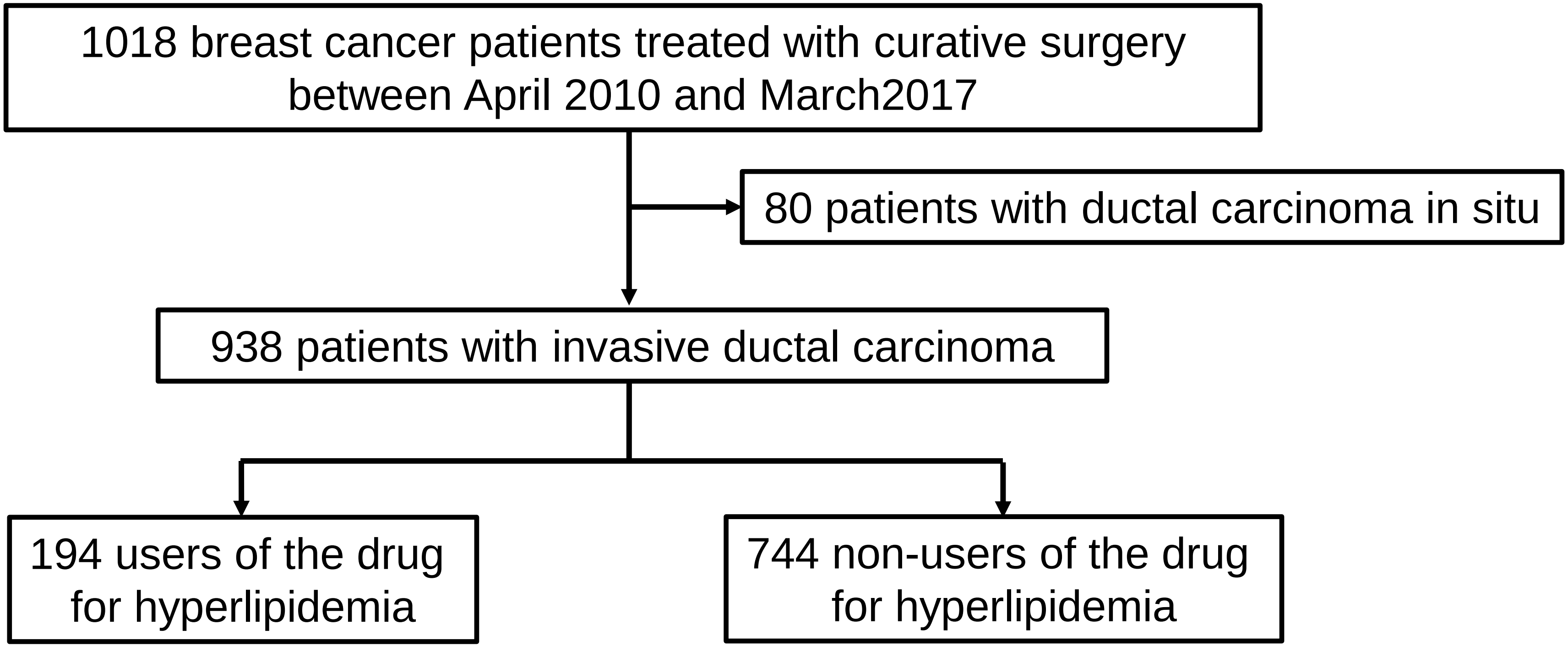

Supplementary Fig. S2 Consort diagram. A total of 1018 patients were diagnosed with breast cancer and underwent curative surgery. We excluded 80 patients with ductal carcinoma in situ, and this retrospective study comprised 938 breast cancer patients. Of the 938 breast cancer patients, 194 were receiving treatment for dyslipidaemia.
