# Supplementary material for: Relationship between serum lipid levels and the immune microenvironment in breast cancer patients: a retrospective study
Source: BMC Cancer. 2022 Feb 14;22:167. doi: 10.1186/s12885-022-09234-8 (PMC8842971; doi:10.1186/s12885-022-09234-8)
Supplement: Supplementary file 4 — Additional file 4: Supplementary Figure S4. Overall survival (OS) using Kaplan-Meier method in patients based on users or non-users of drugs for dyslipidemia with different intrinsic breast cancer subtype. Luminal (a), Luminal-human epidermal growth factor receptor 2 (HER2) (b), HER2-enrich (c) and triple-negative breast cancer (TNBC) (d). [file 12885_2022_9234_MOESM4_ESM.pdf]

Supplementary Fig. S4 Goto W. et al.

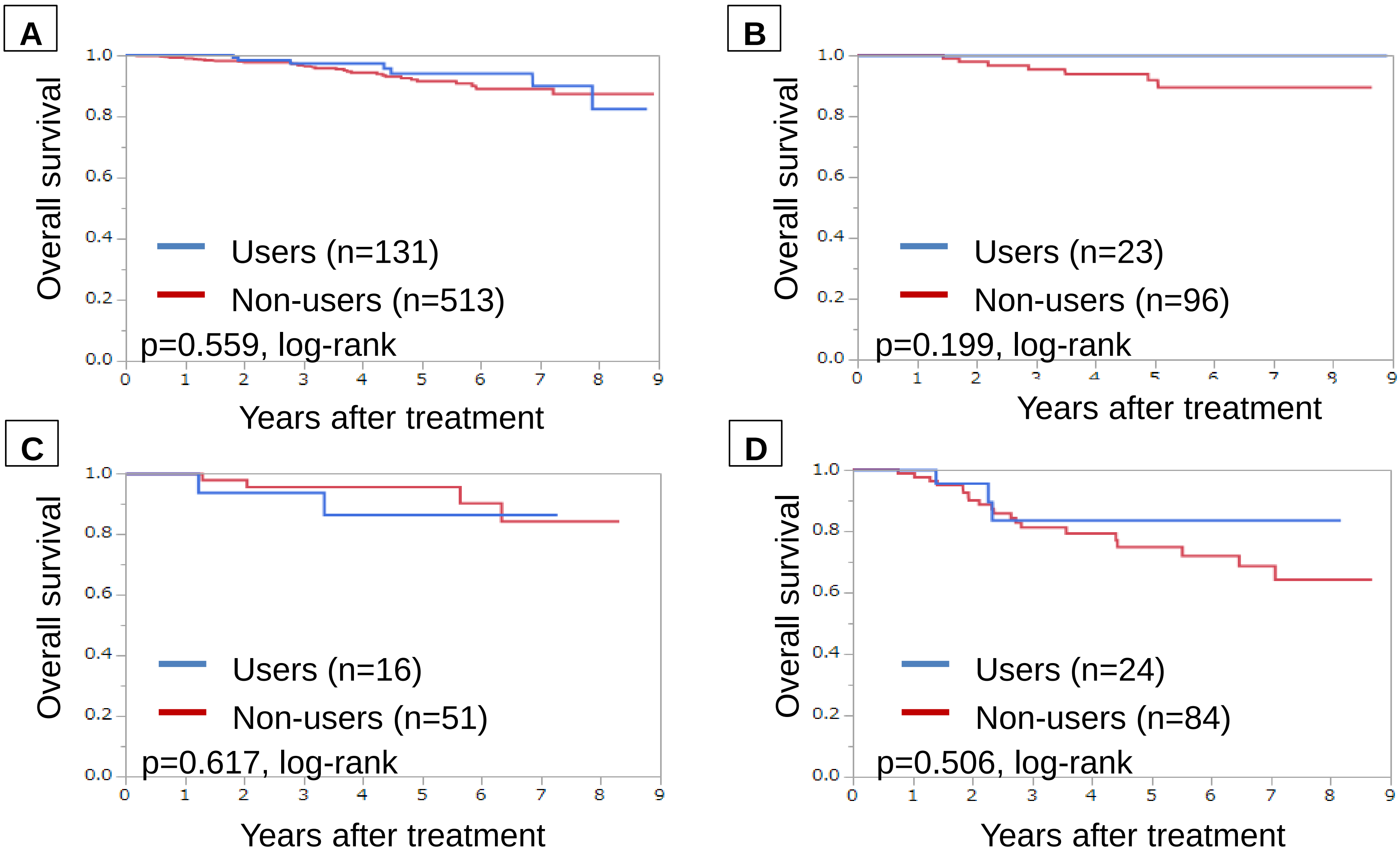

Supplementary Fig. S4 Overall survival (OS) using Kaplan-Meier method in patients based on users or non-users of drugs for dyslipidemia with different intrinsic breast cancer subtype. Luminal (**a**), Luminal-human epidermal growth factor receptor 2 (HER2) (**b**), HER2-enrich (**c**) and triple-negative breast cancer (TNBC) (**d**).
