# Supplementary material for: Relationship between serum lipid levels and the immune microenvironment in breast cancer patients: a retrospective study
Source: BMC Cancer. 2022 Feb 14;22:167. doi: 10.1186/s12885-022-09234-8 (PMC8842971; doi:10.1186/s12885-022-09234-8)
Supplement: Supplementary file 6 — Additional file 6: Supplementary Figure S6. Overall survival (OS) using Kaplan-Meier method in patients treated for dyslipidemia based on control of serum lipid levels with different intrinsic breast cancer subtype. Luminal (a), HER2-enrich (b) and triple-negative breast cancer (TNBC) (c). [file 12885_2022_9234_MOESM6_ESM.pdf]

Supplementary Fig. S6 Goto W. et al.

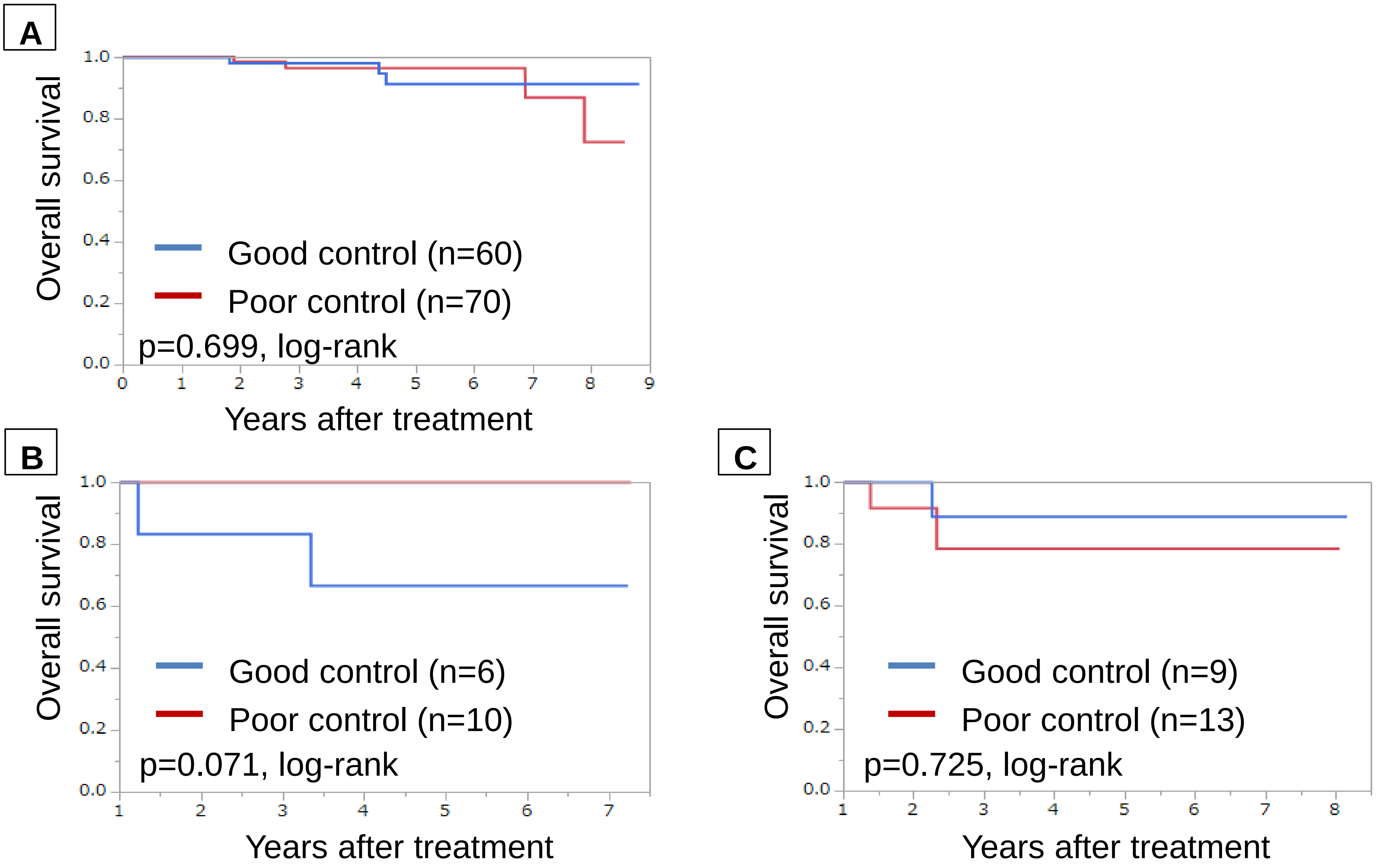

Supplementary Fig. S6 Overall survival (OS) using Kaplan-Meier method in patients treated for dyslipidemia based on control of serum lipid levels with different intrinsic breast cancer subtype. Luminal (**a**), HER2-enrich (**b**) and triple-negative breast cancer (TNBC) (**c**).
