# Supplementary material for: Relationship between serum lipid levels and the immune microenvironment in breast cancer patients: a retrospective study
Source: BMC Cancer. 2022 Feb 14;22:167. doi: 10.1186/s12885-022-09234-8 (PMC8842971; doi:10.1186/s12885-022-09234-8)
Supplement: Supplementary file 7 — Additional file 7: Supplementary Figure S7. Consort diagram. Of the 120 postmenopausal patients with hormone receptor (HR)-positive/human epidermal growth factor receptor 2 (HER2)-negative breast cancer treated for dyslipidaemia, 56 were in good lipid control group. [file 12885_2022_9234_MOESM7_ESM.pdf]

Supplementary Fig. S7 Goto W. et al.

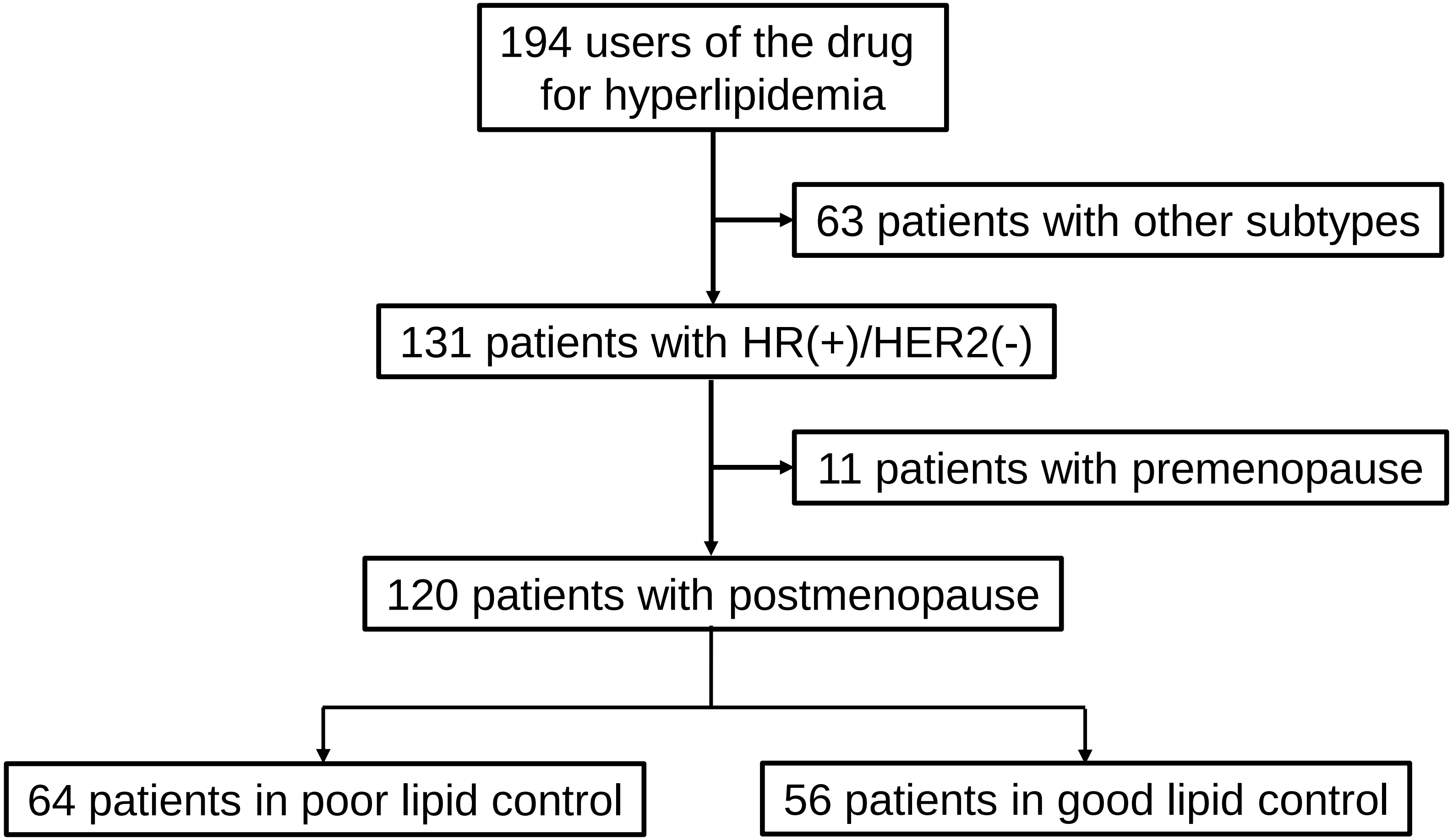

Supplementary Fig. S7 Consort diagram. Of the 120 postmenopausal patients with hormone receptor (HR)-positive/human epidermal growth factor receptor 2 (HER2)-negative breast cancer treated for dyslipidaemia, 56 were in good lipid control group.
