# Supplementary material for: Relationship between serum lipid levels and the immune microenvironment in breast cancer patients: a retrospective study
Source: BMC Cancer. 2022 Feb 14;22:167. doi: 10.1186/s12885-022-09234-8 (PMC8842971; doi:10.1186/s12885-022-09234-8)
Supplement: Supplementary file 8 — Additional file 8: Supplementary Figure S8. Receiver operating characteristic curve analyses of the NLR in postmenopausal hormone receptor (HR)-postive/human epidermal growth factor receptor 2 (HER2)-negative breast cancer patients [file 12885_2022_9234_MOESM8_ESM.pdf]

# Supplementary Fig. S8  Goto W. et al.

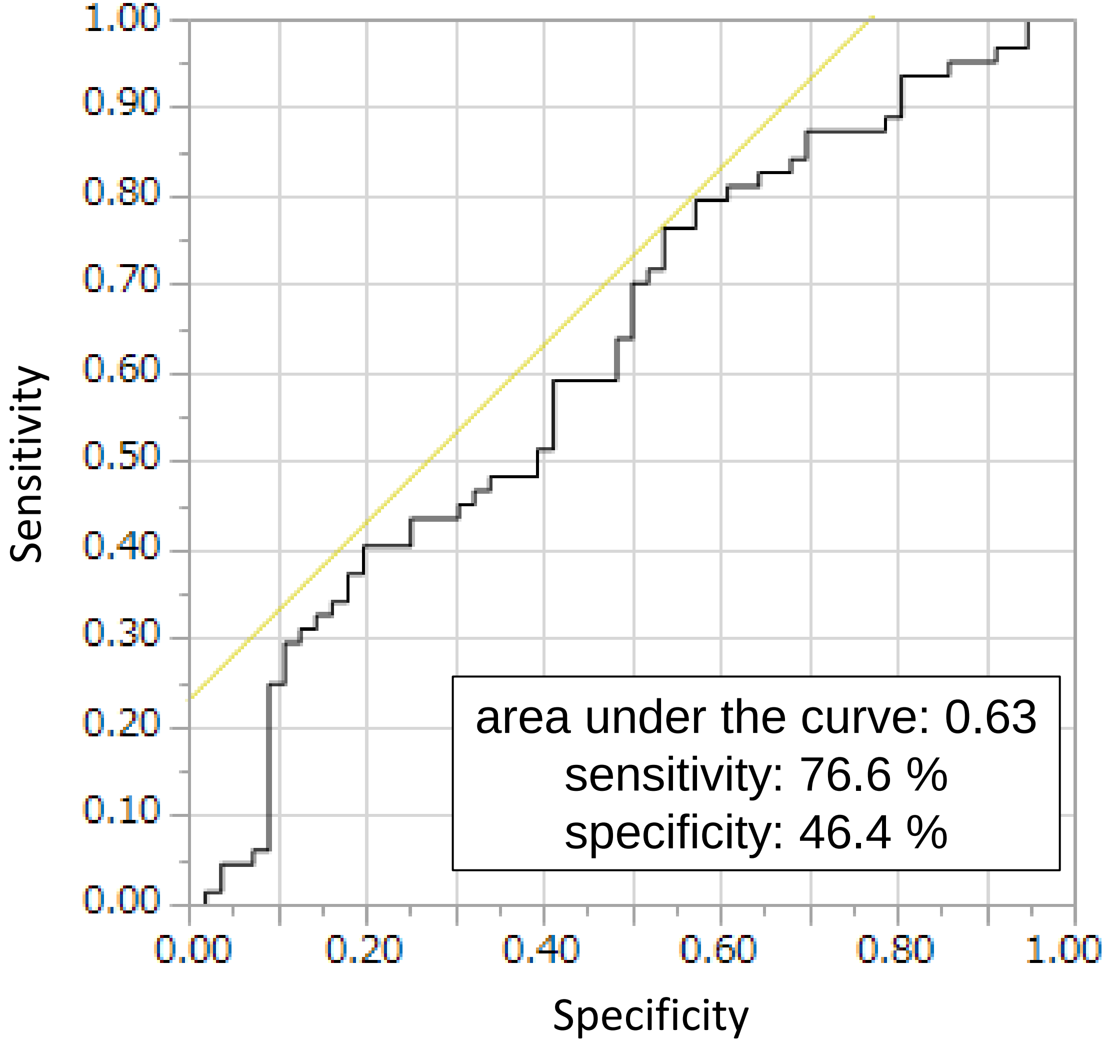


**Supplementary Fig. S8** Receiver operating characteristic curve analyses of the NLR in postmenopausal hormone receptor (HR)-postive/human epidermal growth factor receptor 2 (HER2)-negative breast cancer patients.
