# Supplementary material for: Relationship between serum lipid levels and the immune microenvironment in breast cancer patients: a retrospective study
Source: BMC Cancer. 2022 Feb 14;22:167. doi: 10.1186/s12885-022-09234-8 (PMC8842971; doi:10.1186/s12885-022-09234-8)
Supplement: Supplementary file 9 — Additional file 9: Supplementary Figure S9. Recurrence-free survival (RFS) and overall survival (OS) in postmenopausal hormone receptor (HR)-positive/human epidermal growth factor receptor 2 (HER2)-negative breast cancer patients treated for dyslipidaemia based on preoperative serum lipid levels. Estimated Kaplan-Meier curves of RFS (a) and OS (b). [file 12885_2022_9234_MOESM9_ESM.pdf]

Supplementary Fig. S9 Goto W. et al.

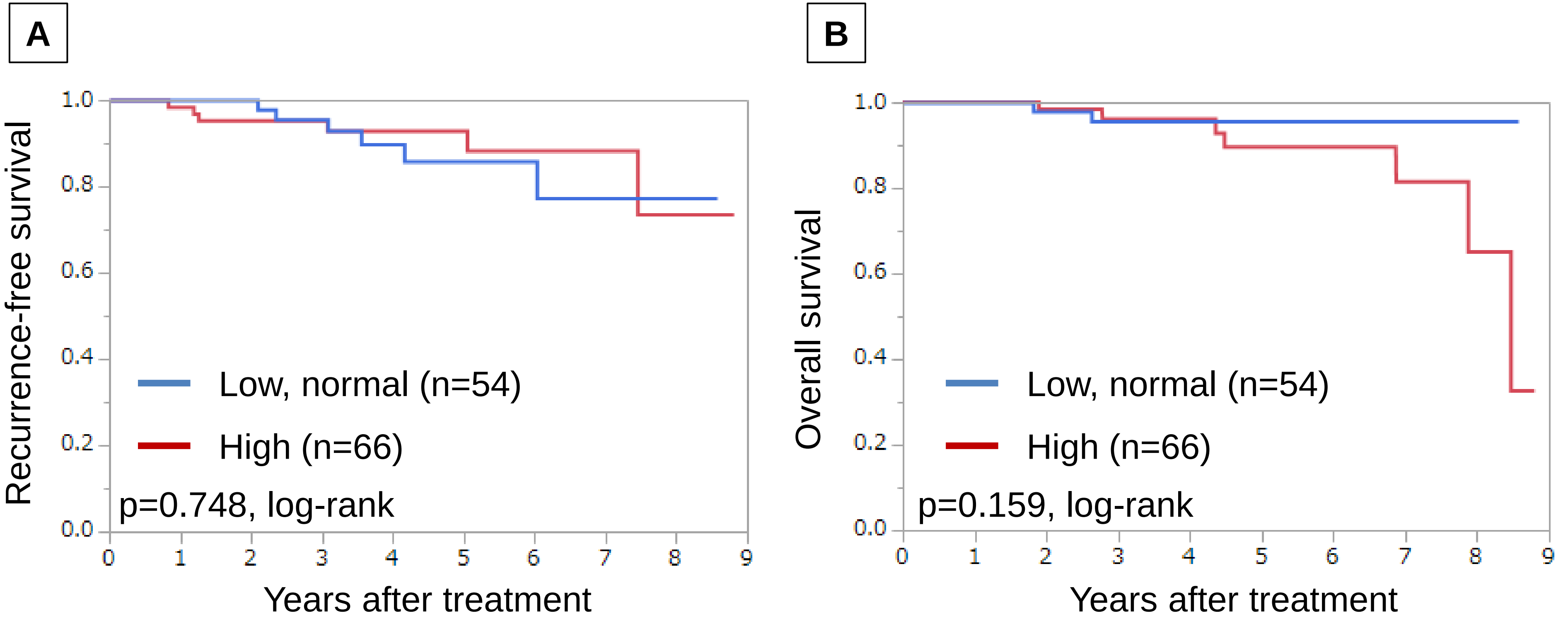

Supplementary Fig. S9 Recurrence-free survival (RFS) and overall survival (OS) in postmenopausal hormone receptor (HR)-positive/human epidermal growth factor receptor 2 (HER2)-negative breast cancer patients treated for dyslipidaemia based on preoperative serum lipid levels. Estimated Kaplan-Meier curves of RFS (**a**) and OS (**b**).
