# Supplementary material for: Relationship between serum lipid levels and the immune microenvironment in breast cancer patients: a retrospective study
Source: BMC Cancer. 2022 Feb 14;22:167. doi: 10.1186/s12885-022-09234-8 (PMC8842971; doi:10.1186/s12885-022-09234-8)
Supplement: Supplementary file 10 — Additional file 10: Supplementary Figure S10. Recurrence-free survival (RFS) using Kaplan-Meier method in postmenopausal hormone receptor (HR)-positive/human epidermal growth factor receptor 2 (HER2)-negative breast cancer patients treated for dyslipidaemia based on statin type. [file 12885_2022_9234_MOESM10_ESM.pdf]

Supplementary Fig. S10 Goto W. et al.

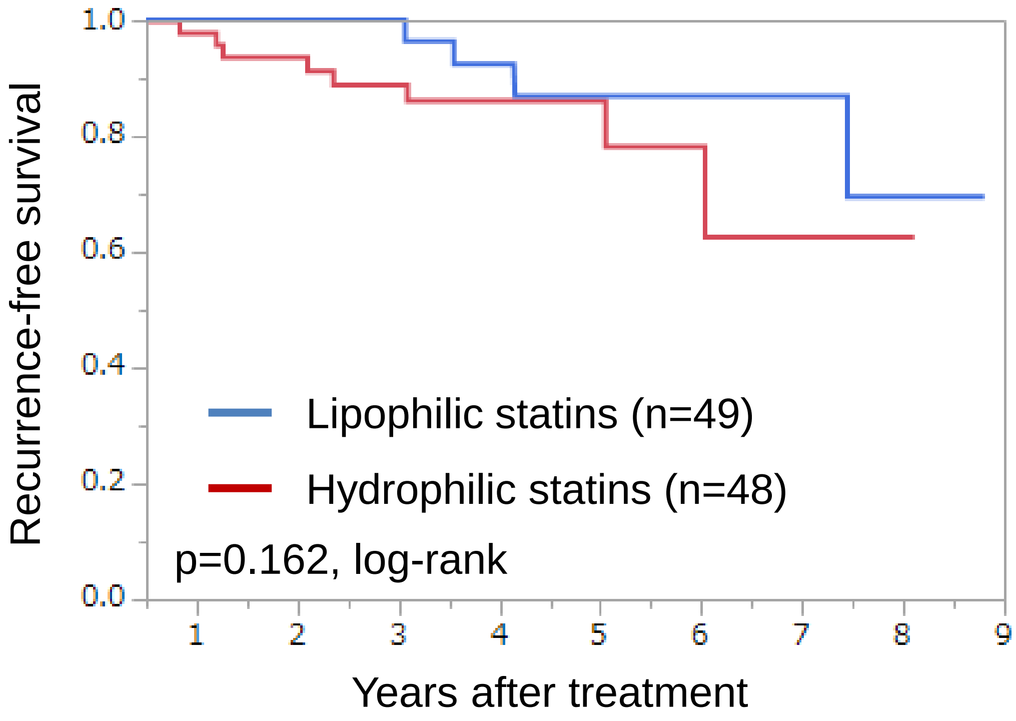

Supplementary Fig. S10 Recurrence-free survival (RFS) using Kaplan-Meier method in postmenopausal hormone receptor (HR)-positive/human epidermal growth factor receptor 2 (HER2)-negative breast cancer patients treated for dyslipidaemia based on statin type.
