# Supplementary material for: Relationship between serum lipid levels and the immune microenvironment in breast cancer patients: a retrospective study
Source: BMC Cancer. 2022 Feb 14;22:167. doi: 10.1186/s12885-022-09234-8 (PMC8842971; doi:10.1186/s12885-022-09234-8)
Supplement: Supplementary file 11 — Additional file 11: Supplementary Table S1. Differences in clinicopathological features due to preoperative serum lipid levels. [file 12885_2022_9234_MOESM11_ESM.docx]

**Supplementary Table S1. Differences in clinicopathological features due to preoperative serum lipid levels.**

|  | Preoperative serum lipid levels (n=120) | |  |
| --- | --- | --- | --- |
|  | High (n=66) | Low, normal (n=54) | p value |
| Age at diagnosis (year) |  |  |  |
| ≤65 / >65 | 21(31.8%) / 45(68.2%) | 21(38.9%) / 33(61.1%) | p=0.447 |
| BMI (kg/m^2^) |  |  |  |
| ≤25.1 / >25.1 | 31(47.0%) / 35(53.0%) | 29(54.7%) / 24(45.3%) | p=0.462 |
| Diabetes |  |  |  |
| Negative / Positive | 47(71.2%) / 19(28.8%) | 38(70.4%) / 16(29.6%) | p=0.920 |
| Adjuvant chemotherapy received |  |  |  |
| No / Yes | 15(22.7%) / 51(77.3%) | 16(29.6%) / 38(70.4%) | p=0.410 |
| Tumor size (cm) |  |  |  |
| ≤2 / >2 | 38(57.8%) / 28(42.2%) | 34(62.5%) / 20(37.5%) | p=0.579 |
| Lymph node status |  |  |  |
| Negative / Positive | 46(69.7%) / 20(30.3%) | 39(72.2%) / 15(27.8%) | p=0.841 |
| NLR (preoperative) |  |  |  |
| High / Low | 41(62.1%) / 25(37.9%) | 37(68.5%) / 17(31.5%) | p=0.565 |
| TILs |  |  |  |
| Low / High | 50(80.6%) / 12(19.4%) | 33(61.1%) / 21(38.9%) | p=0.024 |

BMI, body mass index. NLR, neutrophil-to-lymphocyte ratio. TILs, tumor-infiltrating lymphocytes.
